# Public Response to Community Mitigation Measures for Pandemic Influenza

**DOI:** 10.3201/eid1405.071437

**Published:** 2008-05

**Authors:** Robert J. Blendon, Lisa M. Koonin, John M. Benson, Martin S. Cetron, William E. Pollard, Elizabeth W. Mitchell, Kathleen J. Weldon, Melissa J. Herrmann

**Affiliations:** * Harvard School of Public Health, Boston, Massachusetts, USA; † Centers for Disease Control and Prevention, Atlanta, Georgia, USA; ‡ International Communications Research, Media, Pennsylvania, USA

**Keywords:** disease outbreaks, pandemics, human influenza, surveys, public opinion polls, community participation, health knowledge, attitudes and practices, program acceptibility, public policy, policy review

## Abstract

We report the results of a national survey conducted to help public health officials understand the public’s response to community mitigation interventions for a severe outbreak of pandemic influenza. Survey results suggest that if community mitigation measures are instituted, most respondents would comply with recommendations but would be challenged to do so if their income or job were severely compromised. The results also indicate that community mitigation measures could cause problems for persons with lower incomes and for racial and ethnic minorities. Twenty-four percent of respondents said that they would not have anyone available to take care of them if they became sick with pandemic influenza. Given these results, planning and public engagement will be needed to encourage the public to be prepared.

## Abstract 2

Results from a national survey indicated that most persons would follow public health officials’ guidelines.

Scientists and policymakers are concerned about the emergence of an influenza pandemic for which we will have neither a strain-specific vaccine nor sufficient antiviral medications at the onset of the outbreak. In February 2007, the Community Strategy for Pandemic Influenza Mitigation was issued; it describes the early, targeted, and layered use of nonpharmaceutical interventions, coupled with specific uses of antiviral influenza medications, to reduce transmission of pandemic influenza and mitigate the disease (*1*).

Researchers differ over the potential effectiveness of such community mitigation measures. Evidence to determine the best strategies for protecting persons during a pandemic is limited. Several studies based on findings from mathematical models and historical analyses suggest that early implementation of multiple measures, such as social distancing, school closures, and isolation of sick persons, may be effective in reducing the transmission of the virus (*2*–*6*). Other researchers cite uncertainty (*7*) or believe such measures may not be effective (*8*,*9*).

Community mitigation interventions include 1) isolation and treatment with influenza antiviral medications of all persons with confirmed or probable pandemic influenza; 2) voluntary home quarantine of and provision of antiviral medications as prophylaxis to members of households with persons with confirmed or probable influenza (if sufficient quantities of antiviral medications exist and a feasible means of distribution is in place); 3) dismissal of students from schools and closure of childcare facilities along with preventing the recongregation of children and teenagers in community settings; and 4) social distancing of adults in the community and workplace, which may include cancellation of large public gatherings and possible alteration of workplace environments and schedules to decrease social density. A great deal of cooperation from the public would be required to successfully implement community mitigation measures during a pandemic. Public reaction to an unfamiliar crisis is obviously difficult to predict. However, by using surveys that describe hypothetical scenarios, we can elicit potential responses of persons in these situations. Public opinion and input can help inform policy decisions and provide information about realistic expectations for mitigation measures before a public health emergency arises (*10*). This survey was conducted to gauge public reaction to social distancing and other nonpharmaceutical interventions that may be used during a severe pandemic.

## Methods

Data reported here are derived from a survey by the Harvard School of Public Health Project on the Public and Biological Security. The survey was ≈20 minutes long and consisted of 85 questions. International Communications Research conducted the survey from September 28 through October 5, 2006.

The survey was conducted in English and Spanish with a representative national sample of 1,697 adults >18 years of age, including an over-sample of adults who had children <18 years of age in their households. Altogether, 821 such adults with children were interviewed. In the overall results, this group was weighted to its actual proportion of the total US adult population (cooperation rate was 75%; response rate was 36% [*11*]). Common methods for media and preelection surveys were used, and relied on weighting of the data to ensure representativeness. More information about the survey methods and complete question wordings is available in the Technical Appendix.

Surveys like this one, and others that would be conducted as part of a series in the event of a pandemic influenza, can provide technical assistance to public health officials by monitoring the response of the public to the evolving health threat posed by such an outbreak. In a public health emergency, surveys would have to be conducted with short field periods to enable rapid measurement of how the public reacts to a particular set of circumstances. These rapid cycle surveys would make it possible to provide timely information to public health officials and to ensure a quick response.

Getting survey results to public health officials in real time creates a situation similar to that of preelection polling (i.e., specific events can change the behavior and beliefs of many persons in a relatively short timeframe). National polling organizations that engage in preelection surveys use shorter field periods, which provide more up-to-date information but yield lower response rates than surveys conducted over longer time periods (*12*). Forecasts of voters’ choices in preelection polls have shown that outdated information may introduce more errors into predictions of results than low response rates do (*13*).

Independent studies have shown that the results of statistically weighted data from shorter duration surveys are similar to those based on the higher response rate in surveys of long duration and can be used without an unacceptable risk for bias (*14*–*18*). Nonresponse in telephone surveys produces some known biases in survey-derived estimates because participation tends to vary for different subgroups of the population. To compensate for these known biases, sample data are weighted to the most recent Census data available from the Current Population Survey for sex, age, race, region, and education (*19*). Other techniques, including random-digit dialing, replicate subsamples, callbacks staggered over times of day and days of the week, and systematic respondent selection within households, are used to ensure that the sample is representative.

Possible sources of nonsampling errors for this survey include nonresponse bias, as well as specific wording of questions and the order in which questions are asked. The margin of error for the total sample was ±2.4%. To examine differences among subgroups, we compared responses by testing for differences in proportions, taking into account the effect of the study’s design (*20*).

Because many of the respondents may not have been familiar with pandemic influenza, they were first presented with a descriptive hypothetical scenario: “Now I want to ask you some questions about a possible outbreak in the United States of pandemic flu, a new type of flu that spreads rapidly among humans and causes severe illness. Currently there have not been any cases of pandemic flu in the United States. However, imagine that there was a severe outbreak in the United States, possibly in your community. A lot of persons were getting sick from the flu and the flu was spreading rapidly from person to person.” This scenario was intentionally designed to describe a severe situation without being overly alarming. Respondents were then asked how they would respond to and be affected by the circumstances that would arise from such an outbreak. The small proportion of the respondents who said they would be unable to cooperate with public health authorities could be translated into millions of persons who would have difficulty.

## Results

### Familiarity with Pandemic Influenza

To determine whether respondents understood what was meant by pandemic influenza, the survey asked how familiar the respondents were with the term (it is unfamiliar to most Americans). Forty-one percent said they knew what the term meant. Thirty-three percent reported that they had heard of the term but did not know what it meant, and 25% had never heard of pandemic flu (Technical Appendix).

### Ability to Stay Home

Respondents were asked about their ability to comply with public health recommendations during an influenza pandemic; 94% said they would stay at home, away from others, for 7–10 days if they had pandemic flu. In addition, 85% said all members of their household would stay at home for the same period if a member of their household were sick (Table 1; Technical Appendix).

**Table 1:** Responses to questions about ability to stay home during an influenza pandemic*

Question	Possible responses		
	Yes	No	Don't know/refused to answer
Would stay at home for 7–10 d if public health officials recommended because you had flu	94	4	2
You and all members of household would stay at home for 7–10 d if public health officials recommended because a member of household had flu	85	12	4
If public health officials recommended, would be able to take care of sick household member for 7–10 d at home	85	13	2
If stayed at home with sick household member, would be worried about getting sick yourself	76	22	2
Have someone who could care for you at home if you were sick	73	24	4
	Likely	Unlikely	Don't know/refused to answer/NA
You or a member of your household might lose pay and have money problems	48	50	1
You or a member of your household might have a hard time being stuck at home for so long	46	54	1
You might not be able to get baby formula, diapers, or other important things for a baby in your household†	45	53	1
You or a member of your household might be unable to get the health care or prescription drugs that you need	43	55	2
You might not be able to get care for a disabled person in your household‡	36	48	15
You might not be able to get care for an older person in your household§	35	51	15
You might have difficulty taking care of the (child/children) <5 y in your household¶	32	67	1
You or a member of your household might lose your job or business as a result of having to stay home#	27	71	2

*From the Harvard School of Public Health, Pandemic Influenza Survey, 2006. Numbers represent percentage of responses to each question. NA, not applicable. †Among respondents with major responsibility for children <2 y (n = 174). ‡Among respondents in households with disabled person (n = 470). §Among respondents in households with persons >65 y (n = 408). ¶Among respondents who have major responsibility for children <5 y (n = 262). #Among employed respondents (n = 1,101).

Eighty-five percent said they would be able to take care of sick household members at home for 7–10 days. However, 76% of respondents worried about getting sick if they cared for a sick household member.

Seventy-three percent said that they would have someone available to take care of them at home if they became sick with pandemic flu and had to remain at home for 7–10 days. However, 24% said they would not have someone available to take care of them. Persons living in households with only 1 adult are far more likely not to have someone available to take care of them (45%) compared with persons from households with >1 adult (17%). Approximately one third of low-income (36%), African-American (34%), disabled (33%), and chronically ill (32%) adults said that they would not have anyone who could take care of them. A substantial proportion of the respondents (from 48% to 71%, depending on the measure) believed that they or a household member would likely experience problems if they had to stay at home for 7–10 days and avoid contact with anyone outside their household (Table 1).

### School Closings

Thirty-nine percent of respondents reported having children <18 years of age living in their household (*21*), including 16% with children 13–17 years of age in the household, 22% with children 5–12 years of age, and 14% with children <5 years of age. Of adults in households that had children <18 years of age, 91% said that they have major responsibility for the children in their household (Technical Appendix).

Respondents were told that to keep pandemic influenza from spreading and to protect the safety of children, some communities might close schools and daycare facilities for some period of time. Although the Community Strategy for Pandemic Influenza Mitigation used the term dismissal from school, the survey used the term school closure. Respondents were also told that the length of time schools and daycares would remain closed would probably be tied to the severity of the pandemic influenza outbreak.

If schools and daycare were closed for 1 month, 93% of adults who have major responsibility for children <5 years of age in daycare or children 5–17 years of age and have at least 1 employed adult in the household thought they could arrange care so that at least 1 employed adult in the household could go to work. Eighty-six percent thought they would be able to do so for 3 months (Table 2). Of those who said they could arrange care for 1 month so that at least 1 adult would be able to work, 87% said they or another family member would be the primary caretakers for children if schools and daycares had to be closed. Of these adults, 64% said they would need little or no help even if children had to be kept at home for a long time. Of those who said they would need a lot or some help, 50% said they would rely most on help from family, 11% on friends or neighbors, and 34% on outside agencies (including government agencies, church and community groups, or voluntary agencies).

**Table 2:** Responses to questions about school closings during an influenza pandemic*

Question	Possible responses		
	Yes	No	Don't know/ refused to answer
If schools/daycare closed for 1 mo, could arrange care so that at least 1 employed adult in household could go to work†	93	5	2
If schools/daycare closed for 3 mo, could arrange care so that at least 1 employed adult in household could go to work†	86	11	3
If schools/daycare closed for 1 mo, at least 1 employed person would have to stay home from work†	60	37	3
Among those who could arrange care so that at least 1 adult in household could go to work if schools closed for 1 mo:			
If schools were closed for 3 mo, would be willing to give school lessons at home‡	95	5	<0.5
Would need help giving school lessons at home	47	53	<0.5
If schools and daycare closed for 1 mo would be able to work from home and take care of children§	25	72	3
If public health officials recommended, could keep children from taking public transportation, going to public events and gathering outside home while schools closed for 3 mo‡	85	13	2
	A lot/some	Little/none	Don't know/ refused to answer
Would need outside help with problems of having to keep children at home†	35	64	1
Children in household get free breakfast or lunch at school or daycare¶	25	74	1
If school/daycare closed for 3 mo, would be problem that children could not get free meals¶	8	91	1

*From the Harvard School of Public Health, Pandemic Influenza Survey, 2006. Numbers represent percentage of responses to each question. †Among respondents who have major responsibility for children <5 y in daycare or children 5–17 y in household and have at least 1 working adult in household (n = 634). ‡Among respondents with major responsibility for children 5–17 y in household (n = 610). §Among employed respondents who have major responsibility for children <5 y in daycare or children 5–17 y in household (n = 537) ¶Among respondents who have major responsibility for children <5 y in daycare or children 5–17 y in household (n = 664).

However, 60% of adults who have major responsibility for children <18 years of age said that at least 1 employed person in the household would have to stay home from work. Of employed persons, 25% who have major responsibility for children <18 years of age in their household said that if schools and daycares closed for 1 month, they would be able to work from home and take care of the children.

If schools were closed for 3 months, 95% of adults with major responsibility for children 5–17 years of age said they would be willing to give school lessons at home. Of those who were willing to do so, 47% thought they would need a lot or some help, although 53% said they would need little or no help.

Among adults with major responsibility for children 5–17 years of age, 85% thought that if schools were closed for 3 months, they would be able to keep their children and teenagers from taking public transportation, going to public events, and gathering outside home while schools were closed. Of adults who have major responsibility for children <5 years of age in daycare or children 5–17 years of age in their household, 25% reported that a child in their household gets free breakfast or lunch at school or daycare. Asked specifically about an outbreak of pandemic influenza, 34% of those whose children get free meals at school (8% of the total who have responsibility for children in this age group) said that if schools and daycare were closed for 3 months, not getting the free meals would be a problem.

### Ability to Stay Home from Work

Sixty-three percent of the US adult population was employed at the time of the survey (*22*). Employed respondents were asked about the problems they might face being out of work for various lengths of time. Most employed persons (74%) believed they could miss 7–10 days of work without having serious financial problems; 25% said they would face such problems. Fifty-seven percent thought they would have serious financial problems if they stayed home for 1 month. Of those surveyed, 76% believed they would have such problems if they stayed home from work for 3 months (Table 3; Technical Appendix).

**Table 3:** Responses to questions about staying home from work during an influenza pandemic*†

Question	Possible responses		
	Yes	No	Don't know/ refused to answer
Ever work from home†	27	73	<0.5
Would be a serious financial problem if had to stay home for work for 7–10 d†	25	74	1
Would be a serious financial problem if had to stay home for work for 1 mo†	57	41	2
Would be a serious financial problem if had to stay home for work for 3 mo†	76	22	2
If had to stay home for 1 mo, would be able to work from home for that long†	29	69	2
If had to stay home for 3 mo, would be able to work from home for that long†	19	78	3
Workplace has plan for outbreak of pandemic flu†	19	63	18
Includes encouraging sick to stay home	16		
Provides information about flu	14		
Provides information on what supplies to have at home	12		
Includes expanding options to work from home	6		
Would stay home if public health official said you should, even if employer told you to come to work†	57	35	9
Are you worried employer would make you go to work if sick during an outbreak†	22	77	2
Worried employer would make you go to work if sick during outbreak†	43	50	7
Would stay home if public health official said you should, even if employer told you to come to work†	57	35	9
If had to stay home from work, would still get paid†	35	42	22

*From the Harvard School of Public Health, Pandemic Influenza Survey, 2006. Numbers represent percentage of responses to each question. †Among employed respondents (n = 1,101).

Of employed respondents, 29% said that they would be able to work from home if they were asked to stay home for 1 month because of a serious outbreak of pandemic flu. Of the low-income workers (<$25,000/y), 13% believe that they would be able to work from home for that long, compared with 44% of high-income workers (>$75,000/y).

Employed respondents were also asked about their employers’ plans and policies for dealing with an outbreak of pandemic flu. Few working persons (19%) were aware of any workplace plan to respond to a serious outbreak of pandemic flu.

Of employed adults, 57% said they would stay home from work if public officials said they should; 35% said they would go to work if their employers told them to report to their jobs. Of employed adults, 22% were worried that, in the event of a serious outbreak of pandemic flu in their community, their employer would make them go to work even if they were sick.

Of employed respondents, 50% believed that their workplace would stay open if there was a serious outbreak of pandemic flu, even if public health officials recommended that some businesses in the community should shut down. Forty-three percent thought that their workplace would shut down.

Of employed respondents, 35% thought that if they stayed home from work, they would still get paid; 42% thought that they would not get paid, and 22% did not know whether they would get paid. Low-income respondents (from households <$25,000/y) were significantly less likely than high-income respondents (from households >$50,000/y) to believe they would still get paid (Table 4).

**Table 4:** Responses to questions about potential problems adhering to public health recommendations, by household income, and race/ethnicity*

Question	Total	Household income					Race/ethnicity		
		<$25K	$25–49.9K	$50–74.9K	>$75K		White (non-Hispanic)	Black (non-Hispanic)	Hispanic
	All respondents								
	n = 1,697	n = 226	n = 366	n = 300	n = 501		n = 1,345	n = 133	n = 114
If public health officials recommended, would not be able to take care of sick household member for 7–10 d at home	13	19†	16†	6	6		12	19	15
Do not have someone who could care for you at home if you were sick	24	36‡	25§	22	15		23	34¶	20
If asked to stay home 7–10 d, likely that:									
You or a member of your household might lose pay and have money problems	48	57§	58§	49§	35		43	68#	66#
You or a member of your household might be unable to get the health care or prescription drugs that you need	43	57‡	43§	38	35		41	52#	49
You or a member of your household might lose your job or business as a result of having to stay home	27	41‡	30§	24§	14		20	41#	53#
	Employed respondents								
	n = 1,101	n = 91	n = 224	n = 224	n = 406		n = 855	n = 87	n = 79
Would be a serious financial problem if had to stay home from work for 7–10 d	25	56‡	29†	15	15		23	20	37#
Would be a serious financial problem if had to stay home from work for 1 mo	57	84‡	69†	50§	37		53	65#	68#
Would be a serious financial problem if had to stay home from work for 3 mo	76	93‡	84†	71	64		74	76	88#
If had to stay home for 1 mo, would not be able to work from home for that long	69	85†	79§	71§	55		67	77	77
If you had to stay away from work, you:									
Would still get paid	35	14	25	47**	51**		39††	29	22
Would not get paid	42	64†	57†	30	18		41	48	55
Don’t know	22	22	18	22	23		20	22	23

*From the Harvard School of Public Health, Pandemic Influenza Survey, 2006. Numbers represent percentage responding “yes” to each question. †Statistically higher proportion than $50–74.9K and >$75K. ‡Statistically higher proportion than $25–49.9K, $50–74.9K, and >$75K. §Statistically higher proportion than >$75K. ¶Statistically higher proportion than whites and Hispanics. #Statistically higher proportion than whites. **Statistically higher proportion than <$25K and $25–49.9K. ††Statistically higher proportion than Hispanics.

### Ability to Cooperate with Other Recommendations

Respondents were given a scenario about an outbreak of pandemic influenza and asked if they would cooperate if public health officials recommended that for 1 month they curtail various activities of their daily lives. The initial response between 79% and 93% (depending on the measure) was that they would cooperate (Table 5; Technical Appendix).

**Table 5:** Responses to questions about other community mitigation strategies*

Question	Possible responses		
	Yes	No	Don’t know/refused/ not applicable
Would follow recommendation if public health officials said for 1 mo you should:			
Avoid air travel	93	5	1
Avoid public events like movies, sporting events, or concerts	92	7	<0.5
Avoid going to malls and department stores	91	9	1
Limit your use of public transportation, buses and trains	89	7	4
Cancel doctor or hospital appointments that are not critical at the time	89	10	1
Reduce contact with people outside your own household as much as possible	88	11	1
Avoid going to church or religious services	82	16	1
Postpone family or personal events such as parties, weddings, or funerals	79	18	3
	Likely	Not likely	Don’t know/refused
Would stay in town or city during serious outbreak if public health officials recommended you do so	90	9	<0.5

*From the Harvard School of Public Health, Pandemic Influenza Survey, 2006. Numbers represent percentage of responses to each question.

### Problems Responding to Recommendations

On several measures, more low-income Americans (those who come from households with an annual income <$25,000/y) than high-income Americans believed they would experience problems responding to public health recommendations. Similarly, on many of these measures a higher proportion of African Americans and Hispanic Americans than whites believed they would experience problems (Table 4). The same holds true for persons who described their own health status as fair or poor (Table 6; Technical Appendix).

**Table 6:** Responses to questions about potential problems adhering to public health recommendations by health, chronic illness, and disability status*

Question	Total (n = 1,697)	Health status							
		Fair/poor (n = 196)	Excellent/very good/good (n = 1,481)		Chronic illness			Disabled	
					Yes (n = 355)	No (n = 1,317)		Yes (n = 323)	No (n = 1,354)
If public health officials recommended, would not be able to take care of sick household member for 7–10 d at home	13	25†	11		16	12		21‡	10
Do not have someone who could care for you at home if you were sick	24	34†	23		32§	22		33‡	22
If asked to stay home 7–10 days, likely that:									
You or a member of your household might lose pay and have money problems	48	55	48		47	49		49	48
You or a member of your household might be unable to get the health care or prescription drugs that you need	43	55†	40		50§	40		48	41
You or a member of your household might lose your job or business as a result of having to stay home	27	38†	25		24	28		31	26

*From the Harvard School of Public Health, Pandemic Influenza Survey, 2006. Numbers represent percentage of responses to each question. †Statistically higher proportion than Excellent/very good/good health status. ‡Statistically higher proportion than those who are not disabled. §Statistically higher proportion than those who do not have a chronic illness.

## Conclusions

If community mitigation measures were instituted for a severe influenza pandemic, most respondents would comply with recommendations but would be challenged to do so if their income or job was severely compromised. Results from this survey were useful in shaping the Community Mitigation Guidance because important information was obtained about public acceptability and key public concerns and challenges.

During a severe pandemic, public health authorities are likely to recommend that all but the sickest persons remain home while ill. Strategic planning by home-health, faith-based, and community organizations; medical providers; and public health agencies about how to coordinate care for those who would have to stay home ill during a pandemic will be essential, particularly for those who live alone.

The resiliency of those who would need to stay home during a pandemic will depend on their level of preparedness. Previous studies on personal preparedness at home have shown that respondents have concerns about having sufficient supplies if asked to stay quarantined at home for a prolonged period of time (*23*). Two recent surveys indicate that many Americans have made no preparations for a public health emergency and most have prepared less than they think they should (*24*,*25*). Careful community planning, including public education and engagement, will be needed to encourage the public to be prepared for an emergency like a pandemic.

Survey results also indicated that most persons were concerned about getting sick themselves if they had to stay at home to care for a household member who was ill with pandemic flu. The public must be given accurate information before and during a pandemic about how to provide at-home care along with precautions that caretakers should follow to protect their own health.

Employers can enable employees to comply with public health recommendations during a pandemic (*26*,*27*). Sick leave and other policies (such as telecommuting, staggered shifts, and other strategies) should promote and create incentives for workers to stay home if they or a household member becomes sick during a severe pandemic or if well, to report to work. Well employees should report to work (especially those in health care and other critical infrastructure jobs) to ensure business continuity and the ability to provide care as needed (*28*). Workers should be aware of their employer’s pandemic preparedness plans and other strategies that will promote social distancing at the workplace during a pandemic. Implementing these measures will help to ensure a safer workplace during a pandemic and will mitigate transmission of disease.

Among the key interventions for potentially reducing transmission of the influenza virus during a pandemic will be to dismiss students from schools, close childcare facilities, and keep children from re-congregating in the community. Depending on the severity of the pandemic, the duration of school dismissal could range from a few weeks up to 3 months. How families would cope with the cascading effects from prolonged cancellation of school classes is a concern. Families could face the problem of serious income loss. Most respondents said that at least 1 employed person would have to stay home from work during a pandemic to care for children. Therefore, employers can identify employees who may need to stay home to care for children and determine in advance if those employees could work from home, work staggered shifts, or be trained to take on other responsibilities, or if other employees can be cross-trained to take on some of those job functions. Employers must be prepared for increased absenteeism related to childcare responsibilities.

Community mitigation measures could cause particular problems for persons from low-income families and for racial and ethnic minorities. With these problems in mind, communities should plan for the needs of vulnerable populations who may be adversely affected during a pandemic. Workers who do not have sick or other leave time available will need support if they have to stay home during a pandemic. Communities should explore alternative ways of replacing school-based services, such as free meals, if schools are unable to provide those services.

These findings can inform planners about what the public may do if a pandemic occurs. However, the public might react differently when the event actually occurs. These results should be interpreted with caution in advance of a severe pandemic that could cause prolonged disruption of daily life and widespread illness in a community. Adherence rates to recommendations might be high during the early stages of a pandemic but results may not be as predictive over the course of several months. We have more confidence in the predictive ability of the survey in areas in which the public has a greater amount of personal experience, e.g., workplace issues, income, and the need for assistance at home.

Willingness to adhere to community mitigation measures may be influenced by the severity of illness persons observe in the community relative to their need for income and the level of community, individual, and family disruption. In addition, public response is likely to be affected by the perceived effectiveness of government and voluntary agencies in dealing with crisis situations. Planning for implementation of community mitigation measures, as well as actions to reduce secondary consequences, are important steps in enhancing adherence to public health recommendations.

The communication resources of government can be scarce during a crisis. Such resources can be used most effectively if there are recent data about what the public needs to learn. This was seen in the cases of severe acute respiratory syndrome and anthrax (*29*). During a pandemic, short-duration rapid-turnaround public surveys can provide timely information to public health officials about the acceptability of recommendations and needed communication to the public if problems are found (*15*). Although the challenge is formidable, our best chances of protecting health and maintaining functioning communities during a pandemic rely on optimal adherence to public health measures and a coordinated response within and between communities.

## Supplementary Material

Technical Appendix
