# Supplementary material for: Public Response to Community Mitigation Measures for Pandemic Influenza
Source: Emerg Infect Dis. 2008 May;14(5):778–86. doi: 10.3201/eid1405.071437 (PMC2600239; doi:10.3201/eid1405.071437)
Supplement: Technical Appendix [file 07-1437_Techapp-s1.pdf]

Technical Appendix

PUBLIC RESPONSE TO COMMUNITY MITIGATION MEASURES FOR PANDEMIC INFLUENZA

The data are derived from the *Pandemic Influenza Survey* by the Harvard School of Public Health Project on the Public and Biological Security. Scientists from the Centers for Disease Control and Prevention (CDC) provided technical assistance. Fieldwork was conducted via telephone for the Project by ICR/International Communications Research of Media (PA) between September 28 and October 5, 2006.

The survey was conducted in English and Spanish with a representative national sample of 1,697 adults age 18 and over, including an over-sample of adults who had children under age 18 in their households. Altogether 821 such adults with children were interviewed. In the overall results, this group was weighted to its actual proportion of the total adult population. The cooperation rate was 75%, and the response rate was 36%.

The survey whose results are reported here did not include interviews with cellphone-only adults, which might be a possible source of non-coverage bias. Estimates from the 2006 National Health Interview Survey suggest that about one in eight American homes have only wireless (mainly cellphone) telephone service. The incidence of cellphone-only households is higher for low-income and young adults.[1] A recent study has shown that when data are weighted demographically, including a cell-only sample with a landline RDD sample produces population estimates that are nearly identical to those from the landline sample alone.[2] However, another

study has shown that even after weighting, landline telephone surveys will underestimate the prevalence of certain health behaviors.[3]

The study used in this paper shows that low-income people are likely to encounter more problems than people from higher-income households. It is likely that these problems would be somewhat larger if cellphone-only households were included. However, even without these households, the results suggest that the problems of low-income people are large enough to warrant attention from public health officials.

The dataset is available by emailing Kathleen Weldon at kweldon@hsph.harvard.edu.

Definition of response and cooperation rates[4]

Response rate - The number of complete interviews with reporting units divided by the number of eligible reporting units in the sample. Response Rate 3 (RR3), the method used in this article, estimates what proportion of cases of unknown eligibility is actually eligible. This estimate is guided by the best available scientific information on what share eligible cases make up among the unknown cases. Examples of unknown eligibility include numbers where the telephone was always busy or there was no answer throughout the interview period, numbers that had an answering machine or technical barrier such as call-blocking (and where it was not possible to tell whether or not the number was at a housing unit), and numbers in the sample that were never called.

Cooperation rate - The proportion of all cases interviewed of all eligible units ever contacted.

Weighting of survey data

The following quotation explains the basic concept of weighting data from a survey. More details about weighting are in the methods section of the article itself.

> To get an estimate from our sample of the population's opinion, we do not give each response equal influence. We need to "weight" the responses of undersampled groups more heavily than those of oversampled groups, thereby estimating the poll responses that would have resulted if the survey respondents had matched the population in their demographic characteristics. Consider a simple case, weighting by sex. In a 1,000-person sample that is going to be adjusted by sex, the weight for women would be the number of women out of 1,000 people in the population divided by the number of women in the sample. Each female response would be weighted by (i.e., multiplied by) this ratio. If, for example, the sample had fewer women than the general population, female responses would be given more weight when estimating the population's characteristics on each question (p. 114).[5]

After weighting, the sample for the survey whose results are reported in this article was 48% male, 52% female, 69% non-Hispanic white, 11% non-Hispanic African American, 13% Hispanic American, 2% Asian American, 60% adults aged 18-49, and 39% adults aged 50+.

References

1. Blumberg SJ, Luke JV. Wireless substitution: early release of estimates from the National Health Interview Survey, January-June 2007. National Center for Health Statistics. 2007

December [cited 2008 January 16]. Available from http://www.cdc.gov/nchs/data/nhis/earlyrelease/wireless200712.pdf

2. Keeter S, Kennedy C, Clark A, Tompson T, Mokrzycki M. What's missing from national landline RDD surveys? The impact of the growing cell-only population. Public Opin Q 2007;71:772-792.

3. Blumberg SJ, Luke JV. Coverage bias in traditional telephone surveys of low-income and young adults. Public Opin Q 2007;71:734-749.

4. American Association for Public Opinion Research. Final dispositions of case codes and outcome rates for surveys, 2006. (Accessed December 13, 2007, at http://www.aapor.org/uploads/standarddefs_4.pdf)

5. Voss DS, Gelman A, King G. A review: pre-election survey methodology; details from eight polling organizations, 1988 and 1992. Public Opin Q. 1995;59:98-132.

<u>Survey Questionnaire and Results (appended starting on the next page)</u>

PANDEMIC INFLUENZA SURVEY
Harvard School of Public Health
Project on the Public and Biological Security

Topline Results

The study was conducted for the Harvard School of Public Health Project on the Public and Biological Security via telephone by ICR/International Communications Research, an independent research company. Interviews were conducted September 28-October 5, 2006, among a nationally representative sample of 1,697 respondents age 18 and older, including an oversample of adults who had children under age 18 in their households. Altogether 821 such adults with children were interviewed. In the overall results, this group was weighted to its actual proportion of the total adult population. The margin of error for the total sample is +/- 2.4 percentage points at the 95% confidence level.

E. RECORD GENDER OF RESPONDENT

	**Male**	**Female**
Total	48	52

A. WORRIES AND PRECAUTIONS

1. How likely do you think it is that there will be cases of bird flu among **birds** in the U.S. during the next 12 MONTHS? Do you think it is very likely, somewhat likely, not too likely, or not at all likely?

	**LIKELY**			**NOT LIKELY**			**Don't**	
	**NET**	**Very**	**Somewhat**	**NET**	**Not too**	**Not at all**	**know**	**Refused**
Total	53	14	39	42	29	13	5	*

2. How likely do you think it is that there will be cases of bird flu among **humans** in the U.S. during the next 12 MONTHS? Do you think it is very likely, somewhat likely, not too likely, or not at all likely?

	**LIKELY**			**NOT LIKELY**			**Don't**	
	**NET**	**Very**	**Somewhat**	**NET**	**Not too**	**Not at all**	**know**	**Refused**
Total	44	10	34	53	34	19	3	*

3. How familiar are you with the term "pandemic flu"? Do you know what this term means, have you heard of it, but are not sure what it means, or have you never heard of the term "pandemic flu" before?

	**Know what the term means**	**Have heard of it but not sure what it means**	**Have never heard of the term**	**Don't know**	**Refused**
Total	41	33	25	1	*

4. Do you take prescription drugs or over the counter medications on a regular or ongoing basis, or not?

	**Yes**	**No**	**Don't know**	**Refused**
Total	57	43	*	*

5. I am going to read a list of health items some people have in their homes. For each one, please say if this is something you currently have or do not have. Do you have (INSERT ITEM)?

a. Medicine for fever, such as acetaminophen or ibuprofen

	**Yes, currently have**	**No, do not have**	**Don't know**
Total	84	16	*

b. A thermometer to measure fever

	**Yes, currently have**	**No, do not have**	**Don't know**
Total	83	17	*

c. Anti-viral medicines such as Tamiflu

	**Yes, currently have**	**No, do not have**	**Don't know**
Total	12	88	1

6. Including yourself, how many adults, 18 or older, are there living in your household?

	**Total**
1	26
2	53
3	13
4	6
5+	2
Refused	*

9. Currently, are you yourself employed full-time, part-time, or not at all?

	Total
EMPLOYED (Net)	63
Employed full time	50
Employed part time	13
Not at all	36
Refused	*

(Asked of total respondents employed full-time or part-time; n= 1,101)
10. Are you self-employed, or do you work for someone else?

	Total
Self employed	16
Work for someone else	84
Don't know	*
Refused	*

9/10 Combination Table

	Total
EMPLOYED (Net)	63
Self employed	10
Work for someone else	53
Not at all	36
Refused	*

(Asked of total respondents employed full-time or part-time; n= 1,101)
11. Do you ever work from home?

	Yes	No
Total	27	73

(Asked of total employed respondents who ever work from home; n= 371)
12. How often do you work from home: all the time, at least once a week, a few days a month, or only occasionally?

	All the time	At least once a week	A few days a month	Occasionally	Don't know	Refused
Total	31	22	9	36	1	*

11/12 Combination Table (Asked of total respondents employed full-time or part-time; n=1,101)

	Total
Work from home (Net)	27
All the time	9
At least once a week	6
A few days per month	3
Occasionally	10
Do not work from home	73

9/11/12 Combination Table

	Total
Employed	63
Work from home (Net)	17
All the time	5
At least once a week	4
A few days per month	2
Occasionally	6
Do not work from home	46
Not employed	36
Refused	*

(Asked of total respondents who have 2 or more adults 18+ in household; n= 1,296)

13. How many (other) adults in your household are employed full-time?

	1	2	3+	None	Refused
Total	57	11	3	30	*

(Asked of total respondents who have 2 or more adults 18+ in household that are not employed full-time or 3 or more adults 18+ live in the household; n=651)

14. How many (other) adults in your household are employed part-time?

	1	2	3+	None
Total	26	4	1	70

9/13/14 Combination Table

	Total
Someone in household is employed	79
Someone in household is employed full-time	73
Someone in household is employed part-time	22
No one in household is employed	21
Don't know if any one in household is employed	*

19. Are there any children under the age of 18 living in your household?

	Yes	No	Refused
Total	38	61	*

(Asked of total respondents with children under 18 in household; n= 821)

20. Are you one of the adults in the household who has a major responsibility for these children?

	Yes	No	Don't know
Total	91	9	*

(Asked of total respondents with children under 18 in household; n= 821)

21. How many children under the age of 18 are living in your household?

	1	2	3	4	5-8	Refused
Total	40	35	16	6	2	1

(Asked of total respondents with 1 or more children under 18 in household; n= 810)

22. How many are children 13 to 17?

	1	2	3	4	None
Total	32	8	2	*	57

(Asked of total respondents with 1 or more children under 18 in household; n= 810)

23. How many are children 5 to 12?

	1	2	3	4	None
Total	34	19	4	1	43

(Asked of total respondents with 1 or more children under 18 in household; n= 810)

24. How many are children under 5?

	1	2	3	None	Refused
Total	23	12	2	63	*

19/21/22/23/24 Combination Table

	Total
Have children (Net)	38
Children 13 to 17 years old	16
Children 5 to 12 years old	22
Children under 5	14
No children	61
Refused	*

(Asked of total respondents who have 1 or more children under 5 years old in the household; n= 274)

28. During the day, who principally takes care of the (child/children) in your household **under age five**?

	Total
Respondent	33
Spouse	24
Another adult family member	12
Teenage family member who lives in household	--
A friend	*
Babysitter or "nanny"	7
A childcare facility	16
Childcare in someone else's home	2
Childcare at work	1
Combination	4
Don't know	*
Refused	--

(Asked of total respondents who have 1 or more children 5 to 12 years old in the household; n= 461)

29. After school, who principally takes care of the (child/children) in your household age **five to twelve**?

	Total
Respondent	54
Spouse	19
Another adult family member	12
Teenage family member who lives in household	3
A friend	1
Babysitter or "nanny"	2
A childcare facility	5
Childcare in someone else's home	1
Childcare at work	--
Combination	4
Child does not go to school	--
Child is home schooled	*
Don't know	1
Refused	*

Now I want to ask you some questions about a possible outbreak in the U.S. of pandemic flu, a new type of flu that spreads rapidly among humans and causes severe illness. Currently there have not been any cases of pandemic flu in the U.S. However, imagine that there was a severe outbreak in the U.S. and possibly in your community and a lot of people were getting very sick from the flu and the flu was spreading rapidly from person to person.

B. SCENARIO: HOME

Public health officials think many people will get sick if there is a severe outbreak of pandemic flu. Those less severely sick would need to be taken care of at home rather than at hospitals. Only the sickest people would be hospitalized. I'm going to ask you some questions about two situations: if you yourself were sick, or if you were taking care of someone in your household who was sick from pandemic flu.

32. If public health officials said you should be prepared to take care of members of your household at home for 7 to 10 days if they become sick, would you be able to do that, or not?

	Yes	No	Don't know	Refused
Total	85	13	1	1

(Asked of total respondents who would be able to care for household members at home for 7 to 10 days; n= 1,491)

33. What if taking care of that person involved keeping him or her isolated from others in a separate room and having only one person take care of the sick person for 7 to 10 days? Would you be able to do that?

32/33 Combination Table

	Total
Able to care for sick household members for 7 to 10 days (Net)	85
Able to keep sick household member isolated	78
Unable to keep sick household member isolated	6
Unable to care for sick household members for 7 to t10 days	13
Don't know	1
Refused	1

(Asked of total respondents employed full-time or part-time; n= 1,101)

34. If someone in your household other than you got sick and had to be cared for at home, would you be able to miss work to care for them, or not?

	Yes	No	Don't know	Refused
Total	84	13	3	1

(Asked of total respondents employed full-time or part-time; n= 1,101)

35. Would you be able to work from home AND be able to take care of them, or not?

	Yes	No	Don't know	Refused
Total	37	60	2	1

36. If you were sick with pandemic flu and you had to remain at home for 7 to 10 days, is there someone who could care for you at home, or not?

	Yes	No	Don't know	Refused
Total	73	24	3	1

37. Suppose you had pandemic flu and health officials recommended that you stay at home, away from other people for 7 to 10 days. Is this something you would do, or not?

	Yes	No	Don't know	Refused
Total	94	4	1	1

38. What if another member of your household was sick from pandemic flu and health officials recommended that YOU and ALL members of your household should stay at home, away from other people for 7 to 10 days? Is this something you and other members of your household would do voluntarily, or not?

	Yes	No	Some will, Some won't	Don't know	Refused
Total	85	9	3	3	1

39. If you stayed at home with a household member who was sick from pandemic flu, how worried would you be that you would get sick from the disease yourself? Would you be very worried, somewhat worried, not too worried, or not at all worried?

	Worried			Not worried				
	NET	Very	Somewhat	NET	Not too	Not at all	Don't know	Refused
Total	76	40	36	22	11	11	2	*

(Asked of total respondents who feel all household members would NOT stay home voluntarily; n = 114)

40. If you had a household member who was sick with pandemic flu and were offered a medicine that would help keep you from getting sick, then would you stay at home for 7 to 10 days as recommended, or not?

	Yes	No	Don't know
Total	75	23	1

38/40 Combination Table

	Total
All household members would voluntarily stay at home for 7-10 days	85
All household members would NOT voluntarily stay at home for 7-10 days (Net)	9
Would stay home with meds	7
Would NOT stay home with meds	2
Some will, some will not	3
Don't know	3
Refused	1

41. If public health officials thought you might have been exposed to pandemic flu and recommended that you stay at home for 7 to 10 days so that you would not expose other people to the disease, is this something you would do, or not?

	Yes	No	Don't know	Refused
Total	86	10	4	*

42. If public health officials recommended that you stay at home for 7 to 10 days to help protect yourself and other household members from being exposed to the disease outside your home, is this something you would do, or not?

	Yes	No	Don't know
Total	87	10	3

43. Here is a list of problems people might have while staying at home in the event of an outbreak of pandemic flu. If you were asked to stay at home for 7 to 10 days and avoid contact with anyone outside your household, how likely do you think it is that each of the following would happen to you or a member of your household? How about (READ ITEM) Do you think that is very likely, somewhat likely, not too likely, or not at all likely?

a. You or a member of your household might be unable to get the health care or prescription drugs that you need

	LIKELY			NOT LIKELY				
	NET	Very	Somewhat	NET	Not too	Not at all	Don't know	Refused
Total	43	20	23	55	23	32	2	*

b. You or a member of your household might have a hard time being stuck at home for so long

	LIKELY			NOT LIKELY				
	NET	Very	Somewhat	NET	Not too	Not at all	Don't know	Refused
Total	46	22	24	54	20	34	1	*

c. You or a member of your household might lose pay and have money problems

	LIKELY			NOT LIKELY				
	NET	Very	Somewhat	NET	Not too	Not at all	Don't know	Refused
Total	48	27	21	50	17	33	1	*

d. You or a member of your household might lose your job or business as a result of having to stay home

	LIKELY			NOT LIKELY				
	NET	Very	Somewhat	NET	Not too	Not at all	Don't know	Refused
Total	27	13	14	71	20	51	2	*

e. You might not be able to get baby formula, diapers, or other important things for a baby in your household **(Among those who have major responsibility for children aged 0 to 2 years old; n= 174)**

	LIKELY			NOT LIKELY				
	NET	Very	Somewhat	NET	Not too	Not at all	Don't know	Refused
Total	45	19	26	53	27	26	1	--

f. You might have difficulty taking care of the (child/children) under age 5 in your household **(Among those who have major responsibility for children under 5 years old; n= 262)**

	LIKELY			NOT LIKELY				
	NET	Very	Somewhat	NET	Not too	Not at all	Don't know	Refused
Total	32	14	18	67	20	47	1	*

g. You might not be able to get care for a disabled person in your household **(Among in households with disabled person; n=470)**

	LIKELY			NOT LIKELY					
	NET	Very	Somewhat	NET	Not too	Not at all	Not applicable	Don't know	Refused
Total	36	16	20	48	16	32	14	1	*

h. You might not be able to get care for an older person in your household **(Among in households with person age 65+; n=408)**

	LIKELY			NOT LIKELY					
	NET	Very	Somewhat	NET	Not too	Not at all	Not applicable	Don't know	Refused
Total	35	14	21	51	18	33	12	3	--

C. SCENARIO: FOLLOWING RECOMMENDATIONS

44. Now I'm going to read you a list of steps that public health officials might advise. This would be to prevent the spread of severe flu and help protect you and your family from catching it. As I read each one, please tell me if you would follow such a recommendation, or not. What if they said that for ONE MONTH you should (INSERT ITEM)? Do you think you would do that, or not?

a. Avoid public events like movies, sporting events, or concerts

	Yes	No	Don't know	Refused
Total	92	7	*	*

b. Avoid going to malls and department stores

	Yes	No	Don't know	Refused
Total	91	9	1	--

c. Postpone family or personal events such as parties, weddings, or funerals

	Yes	No	Don't know	Refused
Total	79	18	3	*

d. Avoid air travel

	Yes	No	Not applicable	Don't know	Refused
Total	93	5	1	*	--

e. Limit your use of public transportation, buses and trains

	Yes	No	Not applicable	Don't know	Refused
Total	89	7	4	*	--

f. Cancel doctor or hospital appointments that are not critical at the time

	Yes	No	Don't know	Refused
Total	89	10	1	--

g. Reduce contact with people outside your own household as much as possible

	Yes	No	Don't know	Refused
Total	88	11	1	--

h. Avoid going to church or religious services

	Yes	No	Don't know	Refused
Total	82	16	1	*

45. Suppose there was a serious outbreak of pandemic flu in your town or city and health officials recommended that you and members of your household stay in your town or city. How likely is it that you would stay in your town or city—very likely, somewhat likely, not too likely, or not at all likely?

	LIKELY			NOT LIKELY			
	NET	Very	Somewhat	NET	Not too	Not at all	Don't know
Total	90	75	15	9	3	6	*

D. SCENARIO: SCHOOL

In order to keep pandemic flu from spreading and to protect the safety of children, some communities may close schools and daycare facilities for some period of time. The length of school and daycare closures would probably be tied to how serious the pandemic flu outbreak is. For instance, if there was a severe epidemic, schools and daycare might be closed for a long period of time.

(Asked of total unemployed respondents who have no other employed adult household members and have major responsibility for household children under 5 years old in daycare or 5 to 17 years old; n= 12)

46. If schools and daycare facilities were closed for ONE MONTH to protect children because of a serious outbreak of the disease, how much of a problem would it be for you to take care of the children in your household for that long? Would it be a major problem, a minor problem, or not a problem?

Insufficient data for analysis.

(Asked of total respondents who have at least one employed adult in household and have major responsibility for household children under 5 years old in daycare or 5 to 17 years old; n=634)

47. If schools and daycare facilities were closed for ONE MONTH to protect children because of a serious outbreak of the disease, would you be able to arrange care for the children so that at least one adult in your family could go to work, or not?

	Yes	No	Depends	Don't know
Total	93	5	1	1

(Asked of total employed respondents or respondents who have another employed adult household member and have major responsibility for household children under 5 years old in daycare or 5 to 17 years old and would be able to arrange care for ONE MONTH allowing one adult to work; n= 600)

48. Who would **mainly** take care of the children who live in your household if schools and daycare were closed?

	Total
Respondent	51
Another family member who lives in home	29
Teenage family member	2
Babysitter or "nanny"	1
A family member who lives outside home	5
Friends	*
Neighbors	--
Someone else	*
Children would take care of themselves	6
Combination	5
Don't know	*
Refused	*

(Asked of total employed respondents or respondents who have another employed adult household member and have major responsibility for household children under 5 years old in daycare or a 5 to 17 years old; n= 634)

49. If schools and daycare were closed for ONE MONTH, how many of the employed people in your household if any do you think would have to stay home from work?

	1	2	3	None	Don't know	Refused
Total	52	8	*	37	2	1

(Asked of total employed respondents or respondents who have another employed adult household member and have major responsibility for children under 5 years old in daycare or 5 to 17 years old and for one month would be able to or are unsure if they could arrange for childcare allowing one adult to go to work; n= 611)

50. What if schools and daycare were closed for THREE MONTHS because there was a severe outbreak of pandemic flu in your community? Would you be able to arrange care for the children who live in your household so that at least one adult in your family could go to work, or not?

(Total employed respondents or respondents who have another employed adult household member and have major responsibility for children under 5 years old in daycare or 5 to 17 years old; n= 634)
47/50 Combination Table

	Total
Could arrange care for three months	86
Could arrange care for one month but not three months	6
Could arrange care for one month but for three months it depends	1
Could not arrange care for one month	5
Don’t know/Depends when asked about care	2

(Asked of total employed respondents or respondents who have another employed adult household member and have major responsibility for children under 5 years old in daycare or 5 to 17 years old; n= 634)

51. What if schools and daycare were closed for THREE MONTHS? How many of the employed people in your household if any do you think would have to stay home from work?

	1	2	3	None	Don't know	Refused
Total	50	7	*	40	2	*

(Asked of total employed respondents or respondents who have another employed adult household member and have major responsibility for children aged 5 to 17; n= 610)

52. If schools were closed for THREE MONTHS because there was a severe outbreak of pandemic flu, would you be willing to give school lessons at home to the children in your household while their schools were closed or not?

	Yes	No	Depends
Total	95	5	*

(Asked of total employed respondents or respondents who have another employed adult household member and have a major responsibility for children aged 5 to 17 who are willing to give school lessons at home; n= 580)

53. How much help do you think you would need in order to do this: a lot, some, only a little, or none at all?

	A lot/Some			Only a little/None at all				
	NET	A lot	Some	NET	Only a little	None at all	Depends	Don't know
Total	47	15	32	53	25	28	*	*

(Total employed respondents or respondents who have another employed adult household member and have major responsibility for children aged 5 to 17;n=610)
52/53 Combination Table

	Total
Willing to give school lessons at home	95
Need a lot of help	14
Need some help	30
Need only a little help	24
Need no help	27
Depends	*
Unwilling to give school lessons at home	5
Depends	*

(Asked of total respondents who have major responsibility for children aged 5 to 17; n= 640)

54. Suppose schools were closed for THREE MONTHS, and to protect the health of children, public health officials recommended that you do not let the children or teenagers in your household take public transportation, go to public events, malls, or parties with large numbers of people, or gather with other people outside the home. Do you think it would be possible to keep the children and teenagers in your household from doing these things for THREE MONTHS?

	Yes	No	Don't know
Total	85	13	2

(Asked of total respondents who have major responsibility for children aged 5 to 17; n = 640)

55. How much outside help do you think you would need in order to deal with the problems of having to stay at home and keep the children at home for a long period of time during a severe outbreak of Pandemic flu? Would you need a lot, some, only a little, or none at all?

	A lot/Some			Only a little/None at all			
	NET	A lot	Some	NET	Only a little	None at all	Don't know
Total	35	10	25	64	28	36	1

(Among those who have major responsibility for children aged 5 to 17 and would need a lot or some outside help to deal with keeping children at home; n=225)

56. Which ONE of the following sources do you think you would rely on most for help? Would you rely on (INSERT ITEM)?

	Total
Government agencies	15
Voluntary agencies	6
Community groups	6
Church groups	7
Family	50
Friends	8
Neighbors	3
Someone else	*
Don't know	4

(Asked of total respondents who have major responsibility for household children under 5 years old in daycare or aged 5 to 17; n= 664)

57. Do any of the children in your household get free breakfast or lunch at school or daycare?

	Yes	No	Don't know	Refused
Total	25	74	1	*

(Asked of total respondents who have major responsibility for household children under 5 years old in daycare or 5 to 17 years old who get free meals at school or daycare; n= 119)

58. If schools and daycare were closed for THREE MONTHS, how much of a problem would it be that these children could not get these free meals at school or daycare? Would it be a major problem, a minor problem, or not a problem?

	PROBLEM			Not a problem
	NET	Major	Minor	
Total	34	13	21	66

(Total respondents who have major responsibility for household children under 5 years old in daycare or 5 to 17 years old; n= 664)
57/58 Combination Table

	Total
Yes, child gets free breakfast or lunch (Net)	25
Major problem if school/daycare closed for three months	3
Minor problem if school/daycare closed for three months	5
Not a problem if school/daycare closed for three months	17
No, child does not get free breakfast or lunch	74
Don't know	1
Refused	*

E. SCENARIO: WORK

(Asked of total respondents employed full-time or part-time; n= 1,101)

59. If public health officials said you should stay home from work, but your employer told you to come to work, would you stay at home or go to work?

	I would stay home	I would go to work	Don't know	Refused
Total	57	35	8	1

(Asked of total respondents employed full-time or part-time; n= 1,101)

60. If the Pandemic flu was very serious and public health officials recommended that some businesses in your community should shut down, do you think your workplace would shut down, or would it stay open?

	Shut down	Stay open	Don't know	Refused
Total	43	50	7	*

(Asked of total respondents employed full-time or part-time; n= 1,101)

61. If you had to stay home for ONE MONTH because of a serious outbreak of Pandemic flu, would you be able to work from home for that long, or not?

	Yes	No	Don't know	Refused
Total	29	69	2	*

(Asked of total respondents able to work from home for ONE MONTH; n= 388)

62. Would you be able to work from home for THREE MONTHS or not?

(Total respondents employed full-time or part-time; n= 1,101)
61/62 Combination Table

	Total
Able to work from home for three months	19
Would be able to work from home for one month but not three	9
Would not be able to work from home for one month	69
Would be able to work for one month but don't know about three month	1
Don't know	2
Refused	*

(Asked of total employed respondents who have major responsibility for household children under 5 years old in daycare or 5 to 17 years old and are able to work from home for one month; n= 192)

63. If schools and daycare were closed for ONE MONTH because of a serious outbreak of Pandemic flu, would you be able to work from home that long while **ALSO** taking care of the children in your household, or not?

	Yes	No	Don't know	Refused
Total	87	12	1	*

(Asked of total employed respondents who have major responsibility for household children under 5 years old in daycare or 5 to 17 years old and are able to work from home for ONE MONTH while also caring for children)

64. Would you be able to do that for THREE MONTHS?

(Total employed respondents who have major responsibility for household children under 5 years old in daycare or 5 to 17 years old; n= 537)

61/63/64 Combination Table

	Total
Would be able to work from home and take care of children for three months	20
Would be able to work from home to take care of children for one month but not three	5
Would NOT be able to work from home for ONE MONTH	72
Don't know/Refused	3

(Asked of total respondents employed full-time or part-time; n= 1,101)

65. If there were a severe outbreak of Pandemic flu in your community and you had to stay away from work, would you still get paid or not, or don't you know?

	Would get paid	Would NOT get paid	Don't know	Refused
Total	35	42	22	*

(Asked of total respondents employed full-time or part-time; n= 1,101)

66. If cases of pandemic flu remained in your community for some time, public health officials might recommend that people stay home from work so they do not catch or spread the disease. How long do you think you could stay home from work before it became a serious financial problem? Would it become a serious financial problem if you stayed out of work for 7-10 days?

(Asked of total employed respondents who could stay out of work or are unsure for 7 to 10 days without it becoming a financial problem; n= 905)

67. How about ONE MONTH? Would that become a serious financial problem?

(Asked of total employed respondents who could stay out of work or are unsure for ONE MONTH without it becoming a financial problem; n= 564)

68. How about THREE months? Would that become a serious financial problem?

(Asked of total respondents employed full-time or part-time; n= 1,101)
66/67/68 Combination table

	Total
Staying out of work for three months would NOT be a serious financial problem	22
Staying out of work for one month would NOT be a serious financial problem but three months would	19
Staying out of work for 7-10 days would NOT be a serious financial problem but one month would	32
Staying out of work for 7-10 days would be a serious financial problem	25
Don't know	2
Refused	*

(Asked of total respondents employed full-time or part-time; n= 1,101)

69. If there were a severe outbreak of Pandemic flu in your community, how worried are you that your employer would make you go to work even if you were sick? Are you very worried, somewhat worried, not too worried, or not at all worried?

	Worried			Not worried				
	NET	Very	Somewhat	NET	Not too	Not at all	Don't know	Refused
Total	22	9	13	77	20	57	1	1

(Asked of total respondents employed full-time or part-time; n= 1,101)

70. Has your workplace developed a plan to respond to a possible outbreak of pandemic flu?

	Yes	No	Don't know	Refused
Total	19	63	18	*

(Asked of total employed respondents whose workplace has developed a workplace flu plan; n= 232)

71. Does that plan include (INSERT ITEM), or not?

a. Encouraging sick employees to stay at home
b. Expanding options to work from home.
c. Providing information regarding what supplies to have in your home.
d Providing information about pandemic flu

(Total respondents employed full time or part time; n = 1,101)
70/71 Combination Table

	Total
Work place has plan (Net)	19
Includes encouraging sick to stay home	16
Includes expanding options to work from home	6
Provides information on what supplies to have at home	12
Provides information about flu	14
Work place has NO plan to respond	63
Don't know	18
Refused	*

F. SOURCES OF INFORMATION

72. If there were an outbreak of Pandemic flu in your community, how much would you trust the following sources to give you useful and correct information about the outbreak? How about (INSERT ITEM)? Would you trust them a lot, some, only a little, or not at all?

a. State public health officials

	A lot	Some	Only a little	Not at all	Don't know	Refused
Total	45	37	12	6	*	*

b. Reporters at newspapers or magazines

	A lot	Some	Only a little	Not at all	Don'	Refused

					t know	
Total	10	37	27	26	1	*

c. Commentators on TV and radio

	A lot	Some	Only a little	Not at all	Don't know	Refused
Total	12	41	26	21	1	*

d. Local, town, city, or county public health officials

	A lot	Some	Only a little	Not at all	Don't know	Refused
Total	39	39	15	6	1	*

e. Your doctor or other health care professional

	A lot	Some	Only a little	Not at all	Don't know	Refused
Total	72	20	4	2	1	*

f. Religious leaders in your community

	A lot	Some	Only a little	Not at all	Don't know	Refused
Total	28	34	17	19	2	*

g. The governor of your state

	A lot	Some	Only a little	Not at all	Don't know	Refused
Total	28	38	17	15	2	*

h. The CDC, or Centers for Disease Control and Prevention

	A lot	Some	Only a little	Not at all	Don't know	Refused
Total	60	28	6	4	1	*

30. If there was such a severe outbreak of pandemic flu in your community, health officials might have to recommend that the community take actions to slow the spread of the disease. Which of the following do you think should be the MOST important priority for public health officials? That these actions (READ FIRST ITEMS? That these actions (READ NEXT ITEMS), which ONE of these do you think should be the MOST important priority for public health officials?

	Total
Treat everyone as equally as possible	31
Protect the health of the greatest number of people	26
Give priority to sick and frail people in getting assistance	25
Aim to preserve essential community services like electricity and law enforcement	11
Do not interfere with the civil liberties or freedoms of people in your community	4
Don't know	2
Refused	*

DEMOGRAPHICS

7. What is your age?

	Total
18-29	22
30-49	38
50-64	23
65+	16
Refused	1

(Asked of total respondents with 2 or more adult household members; n= 1,296)

8. Is anyone (else) in your household age 65 or older?

	Yes	No	Refused
Total	16	83	*

7/8 Combination Table

	Total
Someone in household is 65+	22
No one in household is 65+	77
Don't know/Refused	1

D1. In general, would you say your health is excellent, very good, good, fair, or poor?

	Excellent/Very Good/Good				Fair/Poor				
	NET	Excellent	Very Good	Good	NET	Fair	Poor	Don't know	Refused
Total	85	26	36	23	14	11	3	*	*

15. Are you limited in any way in any activities because of physical, mental, or emotional problems?

	Yes	No	Don't know	Refused

Total	19	80	*	*

16. Is anyone (ELSE) in your household limited in any way in any activities because of physical, mental, or emotional problems? (IF NECESSARY: THIS INCLUDES CHILDREN IN THE HH)

	Yes	No	Don't know	Refused
Total	14	85	*	1

17. Do you now have any health problem that requires you to use special equipment, such as a cane, a wheelchair, a special bed, or a special telephone? (IF NECESSARY: Include occasional use or use in certain circumstances.)

	Yes	No	Refused
Total	8	91	*

15/17 Combination Table

	Total
Respondent has disability	21
Respondent does not have disability	78
Don't know/Refused	*

18. Does anyone (ELSE) in your household now have any health problem that requires them to use special equipment, such as a cane, a wheelchair, a special bed, or a special telephone? (IF NECESSARY: Include occasional use or use in certain circumstances.)

	Yes	No	Don't know	Refused
Total	7	93	*	1

15/16/17/18 Combination Table

	Total
Respondent or other household member has disability	30
No one in household has disability	69
Don't know/Refused	*

D1a. Have you been told by a doctor or health professional that you have any of the following medical conditions: heart or lung disease, asthma, kidney disease, diabetes, or a disease that causes decreased immunity such as cancer or HIV/AIDS?

	Yes	No	Don't know	Refused

Total	22	78	*	1

D1b. Does anyone (else) who lives in your household have any of these conditions?

	Yes	No	Don't know	Refused
Total	16	83	*	1

D1a/D1b Combination Table

	Total
Respondent or other household member has chronic illness	31
No one in household has chronic illness	68
Don't know/Refused	1

D2. Is anyone in your household pregnant?

	Yes	No	Don't know	Refused
Total	3	96	*	1

(Asked of total respondents employed full-time or part-time; n= 1,101)
D3. Do you work in a health-care related job?

	Yes	No	Refused
Total	19	81	1

(Asked of total respondents employed in a health care related job; n= 210)
D4. Does your work involve direct contact in the care of patients, or not?

	Yes	No	Don't know
Total	56	44	*

(Total respondents employed full-time or part-time; n= 1,101)
D3/D4 Combination Table

	Total
Work in healthcare related job	19
Involves direct contact with patients	10
Does not involve direct contact with patients	8
Does not work in healthcare related job	81
Refused	1

D5. Do you provide childcare in your home for any children who are not part of your own household?

	Yes	No	Refused
Total	7	92	1

D6. Are you, yourself, of Hispanic or Latino background, such as Mexican, Puerto Rican, Cuban, or other Latin American background?

	Yes	No	Refused
Total	13	87	1

(Asked of total respondents who have a Hispanic, Latino or other Latin American background; n= 114)

D6a. Are you White Hispanic or Black Hispanic?

	White	Black	Don't know	Refused
Total	65	9	21	5

D6/D7 Race Summary Table

	Total
White	69
Black or African American	11
Asian American	2
Native American	2
Hispanic (Net)	13
White Hispanic	8
Black Hispanic	1
Hispanic unspecified	3
Some other race	2
Don't know	*
Refused	1

Education Summary Table

	Total
High school graduate or less (Net)	49
Less than high school graduate (subnet)	15
None or grade 1-8	2
High school incomplete	14
High school graduate +(subnet)	34
High school graduate	31
Business, tech/vocational school	3
Some college or more (Net)	50
Some college, no 4 year degree	25
College graduate + (subnet)	25
College graduate	16
Post-graduate training	9
Don’t know	*
Refused	1

Income Summary Table

	Total
Less than $40K (Net)	35
Less than $15K	6
$15K but less than $20K	8
$20K but less than $25K	6
$25K but less than $30K	5
$30K but less than $40K	8
Less than $40K unspecified	3
$40K+ (Net)	52
$40K but less than $50K	10
$50K but less than $75K	16
$75K but less than $100K	10
$100K	13
$40K+ unspecified	3
Don’t know	5
Refused	7

D10. RECORD REGION FROM SAMPLE FILE

	Total
Northeast	19
North Central	22
South	36
West	23

D11. RECORD METRO STATUS FROM SAMPLE

	Total
In the Center City of an MSA	29
Outside the Center City of an MSA, but inside that county	20
Inside a Suburban County of the MSA	19
In an MSA that has NO center City	4
Not in an MSA	29
